# Antioxidant Activity of *Pastinaca sativa* L. ssp. *sylvestris* [Mill.] Rouy and Camus Essential Oil

**DOI:** 10.3390/molecules25040869

**Published:** 2020-02-16

**Authors:** Călin Jianu, Ionuț Goleț, Daniela Stoin, Ileana Cocan, Alexandra Teodora Lukinich-Gruia

**Affiliations:** 1 Faculty of Food Engineering, Banat’s University of Agricultural Sciences and Veterinary Medicine “King Michael I of Romania” from Timisoara, Calea Aradului 119, RO-300645 Timisoara, Romania; mucetedaniela@yahoo.com (D.S.); negreaileana@yahoo.com (I.C.); 2 Faculty of Economics and Business Administration, West University of Timișoara, 300233 Timisoara, Romania; ionutgolet@gmail.com; 3 OncoGen Centre, County Hospital "Pius Branzeu", Blvd. Liviu Rebreanu 156, RO-300736 Timisoara, Romania; gruia_alexandra@yahoo.com

**Keywords:** essential oil, wild parsnip, *Pastinaca sativa* L. ssp. *sylvestris* [Mill.] Rouy and Camus, antioxidant activity

## Abstract

In the last decade, there has been growing interest in the food industry in replacing synthetic chemicals with natural products with bioactive properties. This study’s aims were to determine the chemical composition and the antioxidant properties of the essential oil of *Pastianica sylvestris*. The essential oil was isolated with a yield of 0.41% (*w*/*v*) by steam distillation from the dried seeds and subsequently analysed by GC-MS. Octyl acetate (78.49%) and octyl hexanoate (6.68%) were the main components. The essential oil exhibited an excellent activity for the inhibition of primary and secondary oxidation products for cold-pressed sunflower oil comparable with butylated hydroxyanisole (BHA) and butylated hydroxytoluene (BHT), which were evaluated using peroxide and thiobarbituric acid values. The antioxidant activity of the essential oil was additionally validated using DPPH radical scavenging (0.0016 ± 0.0885 mg/mL), and *β*-carotene-linoleic acid bleaching assays. Also, the amounts of total phenol components (0.0053 ± 0.0023 mg GAE/g) were determined.

## 1. Introduction

Lipid oxidation and the decomposition of oxidation products represent the main deterioration processes which result in decreasing shelf-life, nutritional value and the generation of off odours and off flavours, altering the texture and colour of food products [1]. These quality deteriorations cause the rejection of affected foods by consumers. To reduce the rate of auto-oxidation, several techniques can be adopted, such as the prevention of oxygen access by using suitable packaging materials, storage of food products on lower temperatures or inactivation of enzymes catalysing oxidation [2]. However, these techniques are not always practical or economical from the nutritional and technological points of view [3]. Under these circumstances, the usage of food antioxidants (BHA, BHT, and propyl gallate), capable of inhibiting or delaying the lipid oxidation, is highly desirable.

Due to the potential adverse health effects of synthetic antioxidants [4,5,6], and as a result of consumer requests to reduce the usage of synthetic additives over the past four decades, hundreds of essential oils (EOs) have been evaluated to identify suitable and safe sources of natural antioxidants. Different studies have demonstrated EOs’ potential as natural preservatives agents for food [7,8,9] and as possible substitutes for synthetic antioxidants [3,10] in specific areas of food processing where their use is not in contrast with their aroma. Despite this potential as food antioxidants, due to their excellent antioxidant properties, EOs have limited applications as food additives. In the European Union, only rosemary extracts were labelled as food additives (antioxidants) by the European Commission (EC) and assigned the number E392 according to Directives 2010/67/EU and 2010/69/EU [11,12] repealed by Regulation (EC) 231/2012 and 1333/2008 [13,14].

Even though a large number of EOs have been studied over the last decades, some of them have not been sufficiently considered or remain unexplored. Wild parsnip, an herbaceous biennial, within the *Apiaceae* (*Umbelliferae*) family, is one of the less studied species regarding its EO bioactive properties. Four subspecies of wild parsnip are found across Eurasia. *Pastinaca sativa* L. ssp. *sativa* is widely cultivated throughout the Northern Hemisphere; ssp. *urens* [Req. ex Godron] Celak. and ssp. *sylvestris* [Mill.] Rouy and Camus are distributed in several countries, including France, Georgia, Italy, Romania, the Russian Federation, Switzerland, and Ukraine; and ssp. *latifolia* [Duby] DC., is endemic to Corsica [15,16]. The EO isolate from wild parsnip seeds is dominated by the aliphatic esters octyl acetate and octyl butyrate [17], while the root EO contains myristicin and terpinolene as principal components [18]. To the best of the authors’ knowledge, no study investigating the antioxidant proprieties of *P. sylvestris* EO has been reported before.

Our research aims are to determine the chemical composition and the antioxidant properties of the EO of *P. sylvestris* grown wild in Romania in order to identify new sources of natural antioxidants with applicability in the food industry.

## 2. Results and Discussion

### 2.1. Essential Oil Composition

The EO was extracted from *P. sylvestris* seeds with a yield of 0.41% (*w*/*v*). The results obtained by GC-MS analysis are presented in Table 1. Thirty-two compounds were identified, representing 99.68% of the total EO. Octyl acetate (78.49%), octyl hexanoate (6.68%), hexyl butyrate (2.71%) and octyl butyrate (1.82%) were the main components of analysed EO. The literature is limited with regard to the chemical composition of the wild parsnip (*P. sativa*) EO. Carroll et al. reported that aliphatic esters, octyl acetate and octyl butyrate occur as the major components of EOs of the mature seeds of wild parsnip [17]. The presences of these aliphatic esters, octyl butyrate (79.5%) and octyl acetate (0.3%), was also recorded in the Turkish *Pastinaca sativa* subsp. *urens* EO [18]. According to Carroll et al., the production of octyl acetate and octyl butyrate in mature fruits of wild parsnip does not share a common genetic regulation. The two are positively correlated phenotypically [17]. This correlation appears to be due entirely to environmental effects, as there is no genetic correlation, in spite of their origin from a common biosynthetic pathway [19].

### 2.2. Antioxidant Activity

Peroxide value is one of the most common tests used to estimate the primary oxidation of oils and fats and measure the concentration of peroxides and hydroperoxides formed in this stage of lipid oxidation [20]. The evolution of the peroxide value during the storage period (20 days) for the control and treated samples are shown in Table 2. The results of ANOVA analysis applied on peroxide values show highly significant (*p* < 0.001) main effects (antioxidant and time) but also a highly significant (*p* < 0.001) interaction effect. The differences at each testing period, according to the Duncan test, are also shown in Table 2. The best results are obtained by BHT and the sample treated with 300 mg/L EO. The changing position (crossing effect) can partly explain the high significance of the interaction effect. For example, after four days of storage, the sample treated with 300 mg/L EO has a significantly (*p* < 0.001) lower peroxide level than BHT, but after 16 days, BHT has a substantially (*p* < 0.001) lower peroxide level than EO (300 mg/L). In this situation, even if it is not clear which one of the two samples perform better, at least we may conclude that the EO (300 mg/L) has comparable results with BHT. Regarding the BHA, the conclusion is more clear-cut because the sample with EO (300 mg/L) performs significantly better (*p* < 0.01) at three measurement periods, the other three cases show no significant differences.

The synthetic results of linear regression analysis are shown in Table 3. Even if not statistically significant, the control is the only equation with a negative intercept (not shown in Table 3). This interpretation problem can be solved by estimating a quadratic equation that would represent a better fit (R-sq. = 0.99). However, for comparability purposes, the linear equation has been used. As can be seen in the linear estimation, slopes are not significantly different between the five samples, but all of them have slopes that are significantly different from the control (*p* < 0.01). This fact can be explained by the quadratic tendency of peroxide values in the control group. This quadratic tendency also represents a second important explanation for the interaction effect identified in the ANOVA analysis.

The thiobarbituric acid value (TBA) value is an index of lipid oxidation, widely used as an indicator for the evaluation of the degree of secondary lipid oxidation [21,22]. The TBA values of the control and treated samples during the 20 days of storage are shown in Table 4. The results of the general ANOVA model applied to the TBA value are very similar with those for peroxide values: highly significant (*p* < 0.001) main effects (antioxidant and time) and also a highly significant (*p* < 0.001) interaction effect. The differences at each testing period, according to the Duncan test, are also shown in Table 4. The best results are obtained by the EO (200 mg/L) for the zero-days testing period and BHT in the rest. Even if the BHT is relatively stable in the first position regarding the antioxidant efficiency, the changing of the position between the other samples at different time intervals is worth noticing. A promising result from our perspective is that TBA values for the EO (300 mg/L) are slightly better than those of BHA. TBA values for BHA are lower in the case of the zero-days and 16-days measurements, with a statistically significant difference (*p* < 0.01). However, the EO (300 mg/L) performs significantly better (*p* < 0.01) in three cases: at the 4s, 12 and 20 day measurements.

The synthetic results of linear regression analysis are shown in Table 5. The control is again the only equation with a negative intercept (not shown in Table 5). A quadratic equation for the control would lead to R-sq. = 0.99. In the linear estimation, the slope of the control is much higher and significantly different from the five tested antioxidants (*p* < 0.01).

The 1,1-diphenyl-2-picrylhydrazyl (DPPH) radical is a stable radical with a maximum absorbance at 517 nm that can readily undergo reduction by an antioxidant [23]. When a solution of DPPH is mixed with that of a substance that can donate a hydrogen atom, then this gives rise to the reduced form with the loss of this violet colour [24]. Table 6 shows the DPPH free radical scavenging activity of the PEO and synthetic additives used as positive controls. Lower IC_50_ value means higher antioxidant activity. The EO (IC_50_ = 0.0016 ± 0.0885 mg/mL) exhibited higher scavenging ability on DPPH radicals than BHA and BHT.

*β*-carotene-linoleic acid bleaching assay is based on the loss of the yellow colour of *β*-carotene when reacting with the radicals produced by linoleic acid oxidation in an emulsion. The rate of *β*-carotene bleaching can be slowed down in the presence of antioxidants [25]. The relative antioxidative activity (RAA) of the EO was calculated using the formula: RAA = A_EO_/A_BHT_, where A_BHT_ is the absorbance of the BHT (the positive control used) and A_EO_ is the absorbance of the EO. The calculated RAA of the analysed EO is 97.646% ± 0.006% (Table 6). Based on the data known to us, the antioxidant activity of the EO from *P. sylvestris* seed has not been reported in other studies, which does not allow a comparative analysis of the results obtained.

The antioxidant activity of EOs is related to their chemical composition [26]. Generally, the antioxidant properties of EOs are attributed to their main compounds [27]. In our study, the aliphatic esters (Table 1) dominate the chemical composition of the tested EO but possess lower antioxidant effectiveness [28]. Octyl acetate was previously reported to exhibit a moderate [28] to weak antioxidant effect [29], while octyl butyrate did not show any antioxidant activity [29]. On the other hand, between the minor components, we find several compounds, including eucalyptol, beta-pinene, para-cymene, caryophyllene, and limonene, recognized for their antioxidant properties [28]. Based on this information, the antioxidant activity of the EO isolated from *P. sylvestris* seeds probably depends partially on the synergism and additive effects between components.

### 2.3. Total Phenols Content

Previous studies reported the relationship between the presence of phenols and antioxidant potential of the EOs [30,31,32]. The antioxidant activity of the phenols, organic compounds that contain a hydroxyl group bound directly to the aromatic ring, is mainly due to their ability to donate their phenolic hydrogen to lipid free-radicals [1,25]. The analysed EO records a low phenol content (0.0053 ± 0.0023 mg GAE/g) but exhibited high antioxidant activity comparable with BHA and BHT, the synthetic antioxidants used as positive controls. Teixeira et al. also reported high antioxidant activity for the *Mentha pulegium* EO of Portuguese origin that records a low phenol content [33]. The same tendency was reported for several plant extracts such as *Malva blanca*, *Schinus molle*, *Curcuma longa*, *Cyclanthera pedata*, and *Opuntia soehrensii*, which, despite showing a low content of total phenolics, exhibited a high antioxidant activity [34].

These results suggest that other compounds of different polarities, probably released through hydrolysis and other cleavage processes, may also contribute to the recorded antioxidant activity [23,34]. Moreover, heat- and water-induced chemical reactions can also change the activity of a complex extraction system consisting of numerous compounds with different chemical and physical properties [23].

## 3. Materials and Methods

### 3.1. Chemicals

All reagents used were of analytical grade. Thiobarbituric acid (TBA), chloroform, diphenylpicrylhydrazyl radical (DPPH), Folin and Ciocalteu′s phenol reagent (2N), butylated hydroxyanisole (BHA), butylated hydroxytoluene (BHT) and C_8_-C_20_ alkane standard mixture were purchased from Sigma-Aldrich (Germany).

### 3.2. Essential Oil Extraction

Mature seeds of *P. sylvestris* were harvested from the Ludeștii de Jos, Hunedoara County (Coordinates: 45°43′5″ N 23°10′21″ E) in August 2018. After the identification, a voucher specimen (VSNH.BUASTM-122) was deposited in the Herbarium of the Faculty of Agronomy, Banat’s University of Agricultural Sciences and Veterinary Medicine “King Michael I of Romania” from Timisoara. The seeds were dried under natural conditions (protected from sunlight and natural ventilated). The *P. sylvestris* essential oil (PEO) was obtained through steam distillation, according to the method previously described [35]. After decantation, the EO was dried over anhydrous sodium sulphate (Sigma, Germany) and stored in sealed amber vials at −18 °C.

### 3.3. Gas Chromatography-Mass Spectrometry Analysis

The EO sample was diluted in hexane 1:1000 and injected in an HP6890 Gas-Chromatograph coupled with HP5973 Mass Spectrometer. One μL of the sample was injected in splitless mode on a capillary column, Br-5MS, 5% Phenyl-arylene-95% Dimethylpolysiloxane, 30 m length, 0.25 mm internal diameter, 0.25 μm film thickness (Bruker). The gas chromatograph’s oven temperature programme was in a range of 50 to 300 °C with 6 °C/minute, with a final hold of 5 min and a solvent delay of 3 min. The mass spectrometer source was set at 230 °C, MS Quad was set at 150 °C, and the ionization energy was 70 eV. The gas helium flow rate was 1 mL/min. The mass of the compounds scanned was between 50 and 550 amu. The compounds present in the sample were evaluated based on their spectra compared to the mass spectra from the NIST0.2 library (USA National Institute of Science and Technology software). Area percentage was calculated, and a semi-qualitative analysis was made based on their retention times by calculating their retention indices (RIs), based on a calibration curve with a C_8_–C_20_ alkane standard mixture (Sigma-Aldrich, St. Louis, MO, USA). A comparison of their calculated RIs with Adams indices from literature data was made [36].

### 3.4. Antioxidant Activity

#### 3.4.1. Sample Preparation

To assess the antioxidant activities of PEO, cold-pressed sunflower oil acquired from the local market, with an initial peroxide value 1.8 meq kg^−1^, was used in our research. The antioxidant activity of PEO was tested by comparing the activity of two synthetic antioxidants such as BHA and BHT by peroxide and thiobarbituric acid values. The calculated quantities of EO, 100, 200 and 300 mg/L, respectively, were added to 10 mL of cold-pressed sunflower oil. Separately, 200 mg/L of each antioxidant, the maximum amount of BHT and BHA in fats and oils for the professional manufacturer of heat-treated foodstuffs according to Directive 2006/52/EC [37], were added also to 10 mL cold-pressed sunflower oil. Meanwhile, 10 mL of cold-pressed sunflower oil without any additive was used as a control sample.

#### 3.4.2. Peroxide Value

The peroxide values (meq of oxygen kg^−1^) were determined by the potentiometric method according to ISO 27107:2010 [38] every four days. All tests were replicated three times.

#### 3.4.3. Thiobarbituric acid value (TBA)

The test was performed according to the methods previously described [23] with minor modifications. Two g of each of sample was prepared as described according to the peroxide value method and with 5 mL of benzene and 4 mL of thiobarbituric acid (0.67% aqueous) were added. The mixtures thus prepared were shaken for one hour using a mechanical stirrer. After one hour, the supernatant was taken and placed in a boiling water bath for 45 min. The absorbance of the supernatant was measured after cooling at 540 nm with Specord 210 Analytik Jena spectrophotometer. All tests were replicated three times.

#### 3.4.4. Scavenging Effect on 1,1-diphenyl-2-picrylhydrazyl Radical (DPPH)

The EO was analysed regarding free radical scavenging activity using a Brand–Williams’ adapted method [39,40]. Briefly, 100 μL of samples at different concentrations (1.5, 0.75, 0.375, 0.187, 0.093 mg/mL), diluted in methanol, were placed in a 96-well microplate, and then 10 μL of DPPH (1 mg/mL) solution was added. After incubation for 30 min in the dark at room temperature, the absorbance was measured at 515 nm using a spectrophotometer Tecan i-control, 1.10.4.0 infinite 200Pro. BHT and BHA were used as the positive controls, and methanol served as the negative control. An inhibition percentage of the DPPH free radical was calculated after the formula: I% = (A_blank_ – A_sample_/A_blank_) × 100, where A_blank_ expresses the absorbance of the control, and A_sample_ expresses the absorbance of the test sample.

IC50 was obtained using the BioDataFit 1.02 software (Chang Broscience Inc, Castro Valley, CA, USA). All tests were replicated three times.

#### 3.4.5. *β*-Carotene Bleaching Assay

Oxidation scavenging activity of EO sample was performed using the *β*-carotene bleaching method [25]. Briefly, a stock solution of the *β*-carotene-linoleic acid mixture was prepared: 0.5 mg *β*-carotene was dissolved in 1 mL of chloroform, then, 25 µl linoleic acid and 200 mg Tween 40 were added. Chloroform was evaporated entirely by using a vacuum evaporator. Later, 100 mL of distilled water saturated with oxygen was added and shaken vigorously for 2–3 min until an emulsion was formed. Then, 2.5 mL of the solution was transferred into the test tubes with 350 μl of the EO sample (2 g/L concentration). The emulsion system was incubated for up to 48 h at room temperature. The same procedure was repeated for the synthetic antioxidant (BHT), used as positive controls, and a blank probe containing only 350 μl of ethanol. After the incubation period, the absorbances of the mixtures were measured at 490 nm. All tests were replicated three times. The antioxidative capacities of the EO sample were compared with those of blank and BHT.

#### 3.4.6. Determination of Total Phenols

The total phenolic content (TPC) of the EO sample was performed with an adapted Folin–Ciocalteu method, as previously described [40,41] with minor modifications. Briefly, 15 mg of the EO sample was weighted and mixed with 1 mL methanol. The mixture was vortexed and combined in a 1:5 ratio sample with Folin–Ciocalteu reagent (diluted 1:10 in distilled water to obtain a 0.25 N concentration) and left for five minutes in the dark at room temperature; after this, an equal volume of 7.5% sodium carbonate solution with Folin–Ciocalteu reagent was added, mixed, and left for 1 h in the dark at room temperature. The samples were plated in triplicate in 96-well plates, and the absorbance was measured at 725 nm with a spectrophotometer Tecan i-control, 1.10.4.0 infinite 200Pro. TPC was expressed in gallic acid equivalents (mg GAE/g sample) calculated after a propyl gallate calibration curve with concentrations between 0.375 mg/mL to 0.732 µg/mL.

### 3.5. Statistical Analysis

To preserve the comparability of the results, the statistical methodology used in our research was mainly in line with the existing research in the field [42]. To compare the antioxidant activity of PEO with the control sample and the other two standard antioxidants, in the first stage of the analysis, the peroxide and TBA values were analysed using two-way ANOVA with main and interaction effects. The levels of the first factor were represented by control, BHT, BHA, and three concentrations of PEO (100, 200, and 300 mg/L). The second factor (ordered) was represented by the incubation period at six levels expressed in days. The scope of this stage was, in the first place, to test if there were significant overall differences between control, BHT, BHA, and three concentrations of PEO regarding their antioxidant activity. Secondly, overall significant differences between the levels of the incubation period were also tested at this stage. Lastly, the interaction effect between antioxidants and the incubation period was tested.

Because the interaction effect proved to be significant (*p* < 0.001), a more thorough analysis was done in the second stage of our research, to reveal and better describe the type of interaction. More specifically, pairwise comparisons were realized between all the levels of the first factor at each level of the incubation period using the Duncan test [42].

The last stage of our research was focused on the antioxidant dynamic during the incubation period. Considering the incubation period as a covariate, this analysis was performed by using simple and multiple regression analysis. The single regression analysis was used for each antioxidant and the control to test the tendency of antioxidant activity in time (i.e., slope). Differences in slope were tested in the framework of multiple regression with dummy variables, including all antioxidants and the control in one equation. Both linear and quadratic time effects were tested. Each statistical method was applied separately for peroxide and TBA values. Statistical analysis of the data was realized mainly by using R 3.5.3 (*Base* and *Agricolae* packages).

## 4. Conclusions

In summary, the main components in the PEO were octyl acetate (78.49%), octyl hexanoate (6.68%), hexyl butyrate (2.71%) and octyl butyrate (1.82%). PEO recorded significant activity for the inhibition of primary and secondary oxidation products. The antioxidant performance of PEO proved to be statistically significant (*p* < 0.01) in specific situations, such as high concentrations (300 mg/L) and long incubation periods (20 days), but is not limited to these conditions. Moreover, the antioxidant activity of the PEO was also confirmed by DPPH radical scavenging and *β*-carotene-linoleic acid bleaching assays. However, the low amounts of total phenol components determined in the analysed EO suggest that other compounds of different polarities, probably released during the cleavage process, may also contribute to the recorded antioxidant activity. Future studies should evaluate the relationship between antioxidant activity and chemical composition of EOs.

In conclusion, the results suggest that the PEO may represent an alternative to the usage of synthetic antioxidants to extend the shelf life of foods containing oils and fats.

## Figures and Tables

**Table 1:** The chemical composition of the EO extracted from *P. sylvestris* seeds.

No	Common Name	RI ^a^	%
1	iso-propyl iso-valerate	884	tr.
2	Nonane	890	tr.
3	Santolina triene	891	tr.
4	Alpha-pinene	919	0.24
5	3-hexyl hydroperoxide	926	0.11
6	2-hexyl hydroperoxide	934	0.14
7	Alpha-phellandrene	954	0.22
8	Beta-pinene	959	0.87
9	Furan, 2-pentyl	970	0.13
10	Octanal	982	0.82
11	Hexyl acetate	991	0.18
12	Para-cymene	1006	0.06
13	Limonene	1011	0.13
14	Eucalyptol	1014	0.25
15	Butyl 2-methylbutyrate	1022	0.14
16	*n*-octanol	1054	0.57
17	*n*-nonanal	1092	0.27
18	Hexyl isobutyrate	1140	0.26
19	Ethylidenecyclohexane	1146	0.17
20	Hexyl butyrate	1190	2.71
21	Decanal	1207	0.56
22	Octyl acetate	1217	78.49
23	Hexyl 2-methylbutyrate	1240	0.66
24	*n*-hexyl iso-valerate	1247	0.16
25	Thymol	1300	0.07
26	Octyl butyrate	1407	1.82
27	Decanol acetate	1428	0.81
28	Caryophyllene	1439	0.74
29	trans-alpha-Bergamotene	1453	0.94
30	beta-Cubebene	1502	0.56
31	Octyl hexanoate	1601	6.68
32	Chamazulene	1735	0.92
		Total	99.68

^a^ the retention index (RI) was calculated using a homologous series of *n*-alkanes C_8_- C_20_; tr. (trace): <0.05.

**Table 2:** Inhibitory effect of the *P. sylvestris* seeds essential oil on the primary oxidation of sunflower oil measured by peroxide value method (meq of oxygen kg^−1^).

Treatment	0 Days	4 Days	8 Days	12 Days	16 Days	20 Days
Control	1.71 ^a^ (±0.15)	4.4 ^a^ (±0.45)	7.25 ^a^ (±0.25)	19.25 ^a^ (±0.54)	26.07 ^a^ (±0.3)	41.58 ^a^ (±0.74)
BHT	**1.61 ^a^ (±0.15)**	2.8 ^bc^ (±0.08)	4.29 ^de^ (±0.3)	**6.43 ^d^ (±0.46)**	**9.53 ^d^ (±0.28)**	12.31 ^d^ (±0.37)
BHA	1.68 ^a^ (±0.14)	2.9 ^b^ (±0.02)	4.97 ^bc^ (±0.14)	6.86 ^cd^ (±0.09)	10.63 ^c^ (±0.23)	14.58 ^bc^ (±0.28)
EO (100 mg/L)	1.74 ^a^ (±0.09)	4.01 ^a^ (±0.17)	5.2 ^b^ (±0.14)	8.02 ^b^ (±0.2)	13.06 ^b^ (±0.06)	15.09 ^b^ (±0.06)
EO (200 mg/L)	1.72 ^a^ (±0.08)	2.34 ^cd^ (±0.19)	4.56 ^cd^ (±0.15)	7.39 ^bc^ (±0.17)	11.11 ^c^ (±0.32)	13.86 ^c^ (±0.01)
EO (300 mg/L)	1.7 ^a^ (±0.06)	**1.82 ^d^ (±0.02)**	**4.04 ^e^ (±0.08)**	6.93 ^cd^ (±0.06)	10.82 ^c^ (±0.13)	**11.91 ^d^ (±0.23)**

^a, b, c, d, e^—values with different superscript are significantly different (*p* < 0.01) according to Duncan test; lowest values are marked; bolded in grey cells; each value is the Mean ± SD.

**Table 3:** Linear regression analysis of peroxide values with incubation period as independent variable.

Peroxide Value (days)	Control	BHT	BHA	EO		
				100 mg/L	200 mg/L	300 mg/L
R-sq.	0.93	0.97	0.96	0.96	0.96	0.95
Slope	1.97 ^a^	0.54 ^b^	0.64 ^b^	0.69 ^b^	0.64 ^b^	0.58 ^b^
Std. error	0.14	0.03	0.03	0.03	0.03	0.03

^a, b^—slopes with different superscript are significantly different (*p* < 0.01) in multiple regression equation; R-squared, slope and standard error of slope are shown for each single regression equation.

**Table 4:** Inhibitory effect of the *P. sylvestris* seed essential oil on the secondary oxidation of sunflower oil measured by thiobarbituric acid value (TBA) value (μg malondialdehyde g^−1^).

Treatment	0 Days	4 Days	8 Days	12 Days	16 Days	20 Days
**Control**	2.72 ^b^ (±0.06)	8.02 ^b^ (±0.04)	11.14 ^a^ (±0.1)	19.28 ^a^ (±0.03)	40.4 ^a^ (±1.2)	62.8 ^a^ (±0.12)
**BHT**	1.84 ^e^ (±0.03)	**6.76 ^c^ (±0.14)**	**5.93 ^d^ (±0.09)**	**6.93 ^f^ (±0.1)**	**9.73 ^d^ (±0.04)**	**17.2 ^e^ (±0.19)**
**BHA**	2.21 ^d^ (±0.11)	8.51 ^b^ (±0.43)	7.54 ^c^ (±0.3)	14.56 ^b^ (±0.26)	14.67 ^c^ (±0.18)	19.19 ^c^ (±0.12)
**EO (100 mg/L)**	3.00 ^a^ (±0.05)	10.39 ^a^ (±0.32)	8.75 ^b^ (±0.18)	10.45 ^e^ (±0.3)	16.59 ^b^ (±0.17)	22.57 ^b^ (±0.13)
**EO (200 mg/L)**	**1.66 ^f^ (±0.06)**	8.62 ^b^ (±0.25)	8.63 ^b^ (±0.35)	11.63 ^c^ (±0.21)	14.6 ^c^ (±0.17)	18.31 ^d^ (±0.21)
**EO (300 mg/L)**	2.48 ^c^ (±0.04)	6.9 ^c^ (±0.07)	7.73 ^c^ (±0.12)	11.09 ^d^ (±0.22)	16.0 ^b^ (±0.09)	17.25 ^e^ (±0.13)

^a; b; c; d; e; f^—values with different superscript are significantly different (*p*< 0.01) according to Duncan test; lowest values are marked; bolded in grey cells; each value is the Mean ± SD.

**Table 5:** Linear regression analysis of TBA value with storage period as independent variable.

TBA Value (days)	Control	BHT	BHA	EO		
				100 mg/L	200 mg/L	300 mg/L
R-sq.	0.88	0.81	0.92	0.87	0.94	0.97
Slope	2.90 ^a^	0.62 ^b^	0.79 ^b^	0.84 ^b^	0.74 ^b^	0.75 ^b^
Std. error	0.27	0.07	0.06	0.08	0.05	0.03

^a, b^—Slopes with different superscript are significantly different (*p* < 0.01) in multiple regression equation; R-squared, slope and standard error of slope are shown for each single regression equation.

**Table 6:** Total phenolic content, *ß*-carotene bleaching and DPPH radical scavenging activities of the EO extracted from *P. sylvestris* seeds.

Parameter	EO	BHA ^a^	BHT ^b^
DPPH, IC_50_ (mg/mL)	0.0016 ± 0.0885	0.0070 ± 0.0003	0.0091 ± 0.0003
*β*-carotene bleaching (RAAs) ^c^ (%)	97.646 ± 0.006	Nd ^e^	100
Total Phenolic Content, (mg GAE/g) ^d^	0.0053 ± 0.0023	Nd ^e^	Nd ^e^

^a^ BHA—butylated hydroxyanisole; ^b^ BHT—butylated hydroxytoluene; ^c^ RAA—relative antioxidative activity; ^d^ GAE—gallic acid equivalent; ^e^ Nd—not detected.
